# MTA1 aggravates experimental colitis in mice by promoting transcription factor HIF1A and up-regulating AQP4 expression

**DOI:** 10.1038/s41420-022-01052-y

**Published:** 2022-06-28

**Authors:** Ping Li, Dong-Ping Shi, Tao Jin, Dong Tang, Wei Wang, Liu-Hua Wang

**Affiliations:** 1 Department of General Surgery, Huaian Tumor Hospital & Huaian Hospital of Huaian City, Huaian, 223200 P. R. China; 2 Department of Central Laboratory, Huaian Tumor Hospital & Huaian Hospital of Huaian City, Huaian, 223200 P. R. China; 3 Department of Experimental Surgery-Cancer Metastasis, Medical Faculty Mannheim, Ruprecht Karls University, Mannheim, 68167 Germany; 4 Department of General Surgery, General Surgery Institute of Yangzhou, Northern Jiangsu People’s Hospital, Clinical Medical College, Yangzhou University, Yangzhou, 225001 P. R. China

**Keywords:** Genetics, Cell biology

## Abstract

Experimental colitis can persist as a chronic disease, accompanied with an underlying risk of development into colorectal cancer. Metastasis-associated protein 1 (MTA1), as a chromatin modifier, exerts notable association with multiple diseases, including colitis. The current study aims to investigate the mechanism of MTA1/HIF1A/AQP4 axis in experimental colitis in mice. First, experimental colitis mouse models were established using dextran sulfate sodium (DSS) and in vitro colonic epithelial cells FHC inflammation models were with lipopolysaccharide (LPS) for determination of MTA1 and HIF1A expressions. It was found that MTA1 and HIF1A were both highly-expressed in experimental colitis samples. Results of dual-luciferase reporter gene assay and ChIP assay further revealed that MTA1 activated HIF1A, and subsequently induced AQP4 transcription to up-regulate AQP4 in experimental colitis. Following loss- and gain-function, the effects of MTA1/HIF1A/AQP4 axis on apoptosis and viability of colon epithelial cells were detected by a combination of TUNEL staining and flow cytometry, and CCK-8 assay. It was observed that silencing of MAT1 in the FHC and NCM460 cells reduced IL-1β and TNF-α expressions induced by LPS. Meanwhile, AQP4 promoted LPS-induced inflammation, and exacerbated apoptosis of colon epithelial cells and augmented experimental colitis development in mice. In vivo experiments further verified that TGN-020 treatment effectively alleviated DSS-induced experimental colitis in mice and diminished apoptosis of colon epithelial cells. Altogether, MTA1 may promote AQP4 transcription by activating HIF1A, thus exacerbating DSS-induced experimental colitis in mice, which provides a novel direction for the treatment of experimental colitis.

## Introduction

Acute and chronic colitis are episodic, inflammatory conditions that affect millions of people across the globe [1]. Moreover, the pathogenesis of colitis progression is not comprehensively characterized; meanwhile, there has been increasing speculation regarding the role of the gastrointestinal microbiota ecosystem in colitis [2]. Currently, acute infectious colitis is clinically treated with the use of antimicrobials [3]. In contrast, several types of colitis, such as ulcerative colitis, may be very refractory. The last few decades have witnessed the advent of biologics (biological products), which are complex chains of amino acids, sugars, or nucleic acids, including therapeutic proteins, monoclonal antibodies, blood, and blood derivatives. Moreover, biologics have also found extensive use in the treatment of ulcerative colitis. However, biologics usage is accompanied by immense healthcare costs, whereas, adoption of biosimilars has also shown great potential in the treatment of these diseases [4].

Metastasis-associated protein 1 (MTA1) is well-established as a chromatin modifier with notable involvement in the prognoses of numerous malignancies [5]. What’s more, existing evidence further suggests that MTA1 plays a regulatory role in the proliferation and metastasis of colorectal cancer cells [6]. In addition, MTA1 also confers effects on the regulation of inflammatory responses as well as infection [7]. However, the potential and specific mechanism of MTA1 in experimental colitis currently remains elusive. Nevertheless, Overexpression of MTA1 is known to facilitate the expression of hypoxia-inducible factor-1 A (HIF1A) in human non-small cell lung cancer cells [8]. HIF1 is regarded as a heterodimeric transcription factor, which can serve as a mediator of adaptive responses to hypoxia [9]. It is also noteworthy that an existing study identified that the activation of HIF1A in myeloid cells could facilitate dextran sulfate sodium (DSS)-induced colitis development in mice, which is the basis for experimental colitis [10]. Furthermore, HIF1A stabilized by CoCl_2_ was previously uncovered to elevate the levels of aquaporin 4 (AQP4) in human placenta [11]. Aquaporin 4 (AQP4) is considered as a type of transmembrane protein originating from the aquaporin family, and further serves as a vital water channel in the mammalian brain [12]. As previously reported, deficiency in AQP4 can facilitate the attenuation of experimental colitis in mouse models [13]. In light of the aforementioned literature, we speculated that MTA1 could potentially influence the development of experimental colitis with regulation of the HIF1A/AQP4 axis and subsequently performed a series of experiments to validate the same, in an effort to uncover novel diagnostic and therapeutic modalities against experimental colitis.

## Results

### Successful establishment of experimental colitis models

**Fig. 1:**
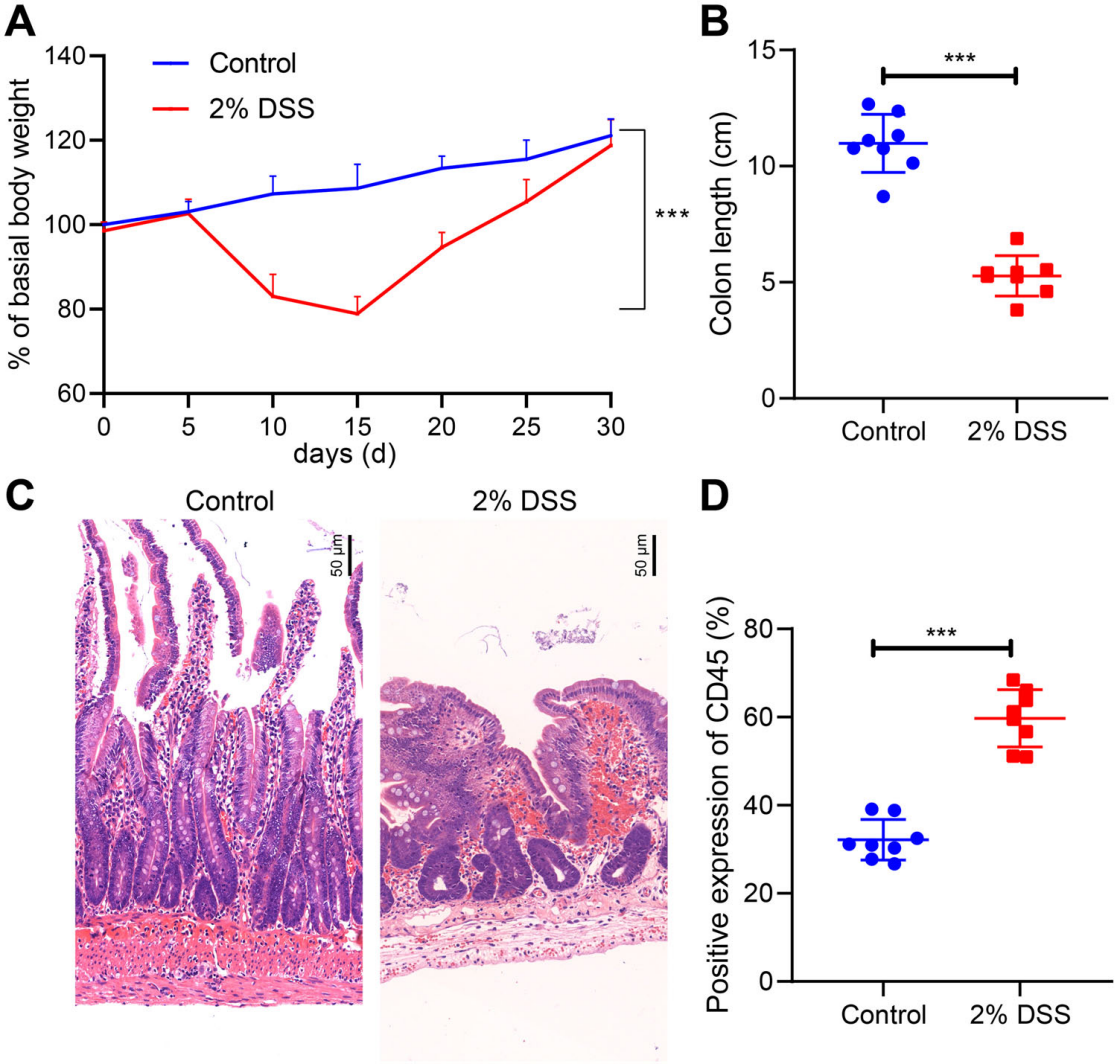
Establishment and identification of experimental colitis in mice. **A** The statistical analysis for 30-day body weight of control mice and 2% DSS-treated mice. **B** Statistics for colon length of control mice and 2% DSS-induced mice. *p < 0.05 *vs*. the control group. **C** HE staining of cross-cut colon tissues in mice (200×). **D** Detection of CD45 positive cell rate in colon tissues of mice. The measurement data were expressed by mean ± standard deviation. Data between two groups were compared by unpaired student’s *t* test, and data at different time points were compared by repeated measures ANOVA, followed by Tukey’s post hoc tests. *n* = 8. ***p < 0.001.

After experimental colitis was induced in mice with 2% DSS, results illustrated a notable reduction in weight of mice (Fig. 1A), accompanied by diarrhea and bloody stools. Following euthanasia, complete colon of the mice was isolated, and it was observed that the colon of mice treated with 2% DSS was shorter compared to that in control mice (Fig. 1B). HE staining also showed evident thickening of the colonic wall in 2% DSS-treated mice, along with severe inflammatory cell infiltration (Fig. 1C). Immunohistochemistry analysis further demonstrated that compared with the control, the concentration of CD45 positive cells was increased in the colon tissue of 2% DSS-treated mice (Fig. 1D).

### MTA1 promotes inflammation in experimental colitis

**Fig. 2:**
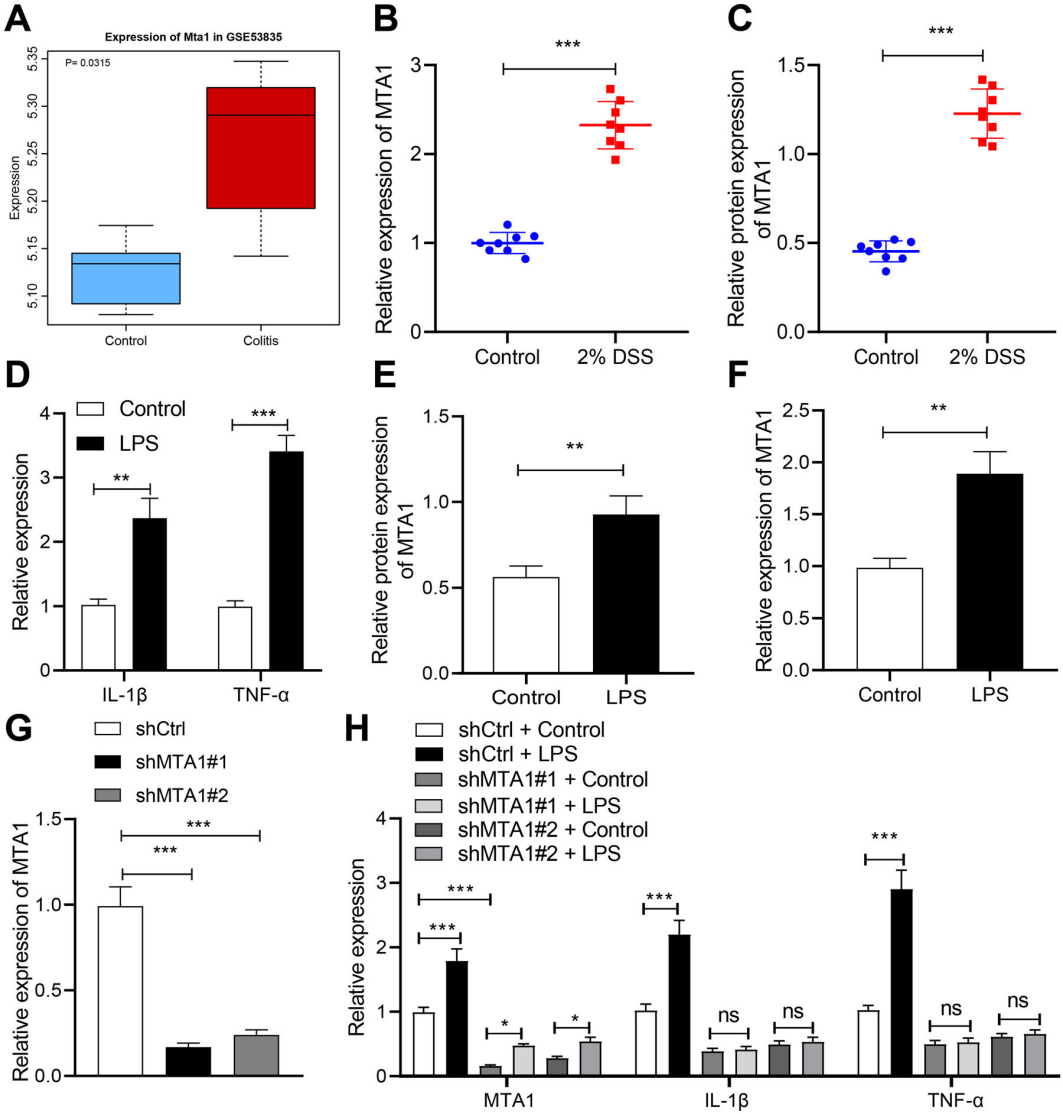
MTA1 promotes the occurrence of experimental colitis. **A** The expression pattern of MTA1 in the control (blue box) and experimental colitis (red box) samples in GSE53835 dataset. **B** The mRNA expression pattern of MTA1 in tissues from control mice and 2% DSS-treated mice analyzed by RT-qPCR, *n* = 8. **C** The protein expression pattern of MTA1 in tissues from control mice and 2% DSS-treated mice detected by western blot, *n* = 8. **D** RT-qPCR to detect the mRNA expression patterns of IL-1β and TNF-α in FHC cells after LPS treatment. **E** The protein expression pattern of MTA1 detected by western blot. **F** The expression pattern of MTA1 mRNA detected by RT-qPCR. **G** RT-qPCR to detect sh-MTA1 silencing efficiency in FHC cells. **H** The expression pattern of MTA1, IL-1β, and TNF-α in FHC cells treated with LPS determined with RT-qPCR. **p < 0.01; ***p < 0.001. The measurement data were expressed as mean ± standard deviation. Data between two groups were compared by the unpaired student’s *t* test, and data among multiple groups were compared by one-way ANOVA, followed by Tukey’s post hoc tests. The experiment was conducted three times independently.

We analyzed the experimental colitis gene expression dataset GSE53835 and found that MTA1 was highly-expressed in experimental colitis (Fig. 2A). mRNA and protein levels of MTA1 were increased after 2% DSS treatment in colon tissues (Fig. 2B, C; Supplementary Fig. 1A). In consistently, MTA1 was up-regulated after lipopolysaccharide (LPS) treatment in FHC cells (Fig. 2E, F; Supplementary Fig. 1B), along with up-regulated interleukin (IL)-1β and tumor necrosis factor (TNF)-α (Fig. 2D).

Next, we adopted sh-MTA1#1 and sh-MTA1#2 to silence MTA1 in the human normal colon epithelial FHC cells. The two independent short hairpin RNAs (shRNAs) achieved good silencing efficiency, while MTA1#1 exhibited superior silencing efficiency, and thus was adopted for subsequent experimentation (Fig. 2G). Following treatment with LPS, there were no alterations in IL-1β and TNF-α levels in response to sh-MTA1#1 and sh-MTA1#2 (Fig. 2H). Meanwhile, identical experimentation with human normal colonic epithelium NCM460 manifested similar results (Supplementary Fig. 2A-E).

### MTA1 upregulates HIF1A and promotes its transcriptional regulation of AQP4

**Fig. 3:**
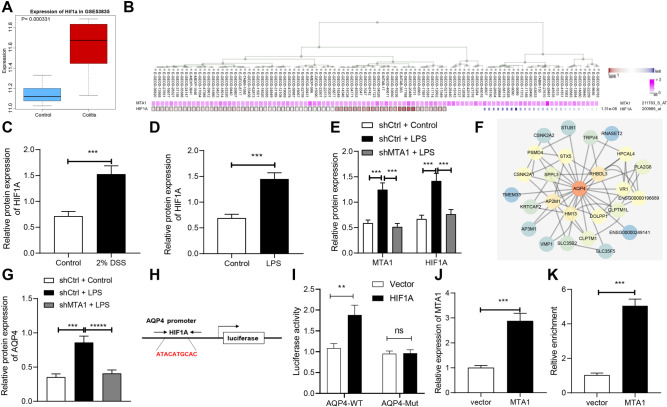
MTA1 upregulates the expression pattern of HIF1A to promote its transcriptional regulation of AQP4. **A** The expression pattern of HIF1A in the control sample (blue box) and experimental colitis sample (red box) in GSE53835 dataset. **B** The co-expression relationship between MTA1 and HIF1A in each dataset analyzed by MEM. (The HIF1A line in the graph was mainly red, indicating that it was mainly positive correlation). **C** Western blot to detect the protein expression of HIF1A in colon tissues after DSS-induced experimental colitis in mice, *n* = 8. **D** The protein expression pattern of HIF1A in FHC cells induced by LPS detected by western blot. **E** The protein expression pattern of HIF1A in MTA1 silenced FHC cells treated with LPS detected by western blot. **F** The interaction gene of AQP4 was obtained from GeneCards and the interaction network was constructed. (The red color of the gene circle suggested that the core degree of the gene in the network was higher; otherwise, the bluer the gene was, the lower the protein expression level was). **G** The expression pattern of AQP4 was detected by western blot after MTA1 silenced FHC cells were treated with LPS. **H** Schematic diagram of the binding site of HIF1A and AQP4. **I** Dual-luciferase reporter gene assay of correlation between MTA1 and HIF1A. **J** RT-qPCR validation of overexpression efficiency of MTA1 overexpression plasmid in FHC cells. **K** The effect of MTA1 overexpression in the enrichment of HIF1A in the promoter region of AQP4 determined with ChIP assay. **p < 0.01; ***p < 0.001. The measurement data were expressed as mean ± standard deviation. Data between two groups were compared by the unpaired student’s *t* test, and data among multiple groups were compared by one-way ANOVA, followed by Tukey’s post hoc tests. The experiment was conducted three times independently.

Furthermore, analyses of GSE53835 indicated that HIF1A was overexpressed (Fig. 3A), while co-expression analysis by MEM exhibited that MTA1 and HIF1A were both markedly co-expressed and shared a positive correlation (Fig. 3B) in experimental colitis. Meanwhile, DSS treatment could radically elevate HIF1A expression in colon tissues, while LPS treatment also enhanced HIF1A expression in FHC cells (Fig. 3C, D; Supplementary Fig. 1C, D). Further, silencing of MTA1 reduced up-regulation of HIF1A in FHC cells induced by LPS (Fig. 3E; Supplementary Fig. 1E).

We subsequently analyzed interaction network of AQP4 using GeneCards database, and identified 25 interacting genes (Fig. 3F). With KOBAS to enrich KEGG pathway, we further detected that the top five enriched pathways of AQP4 and its interaction genes were mitophagy-animal, adherens junction, PD-L1 expression and PD-1 checkpoint pathway in cancer, inflammatory mediator regulation of TRP channels, NF kappa B signaling pathway (Supplementary Table 1). Moreover, existing studies have also determined the association of these pathways with inflammation [14–18]. Subsequent results depicted that LPS elevated AQP4 expression, while further MTA1 silencing inhibited AQP4 expression in FHC cells (Fig. 3G; Supplementary Fig. 1F). As predicted by hTFtarget database, the binding site of HIF1A was identified in the AQP4 promoter (Fig. 3H). In addition, after co-transfection of HIF1A overexpression with AQP4-wild type (WT), the luciferase activity was increased (Fig. 3I). Meanwhile, chromatin immunoprecipitation (ChIP) experimentation illustrated that overexpression of MTA1 increased enrichment of HIF1A in AQP4 promoter region (Fig. 3J, K). Besides, we performed identical experiments on human normal colonic epithelium NCM460, and documented similar results (Supplementary Fig. 3A–C).

### HIF1A promotes experimental colitis by increasing AQP4 expression

**Fig. 4:**
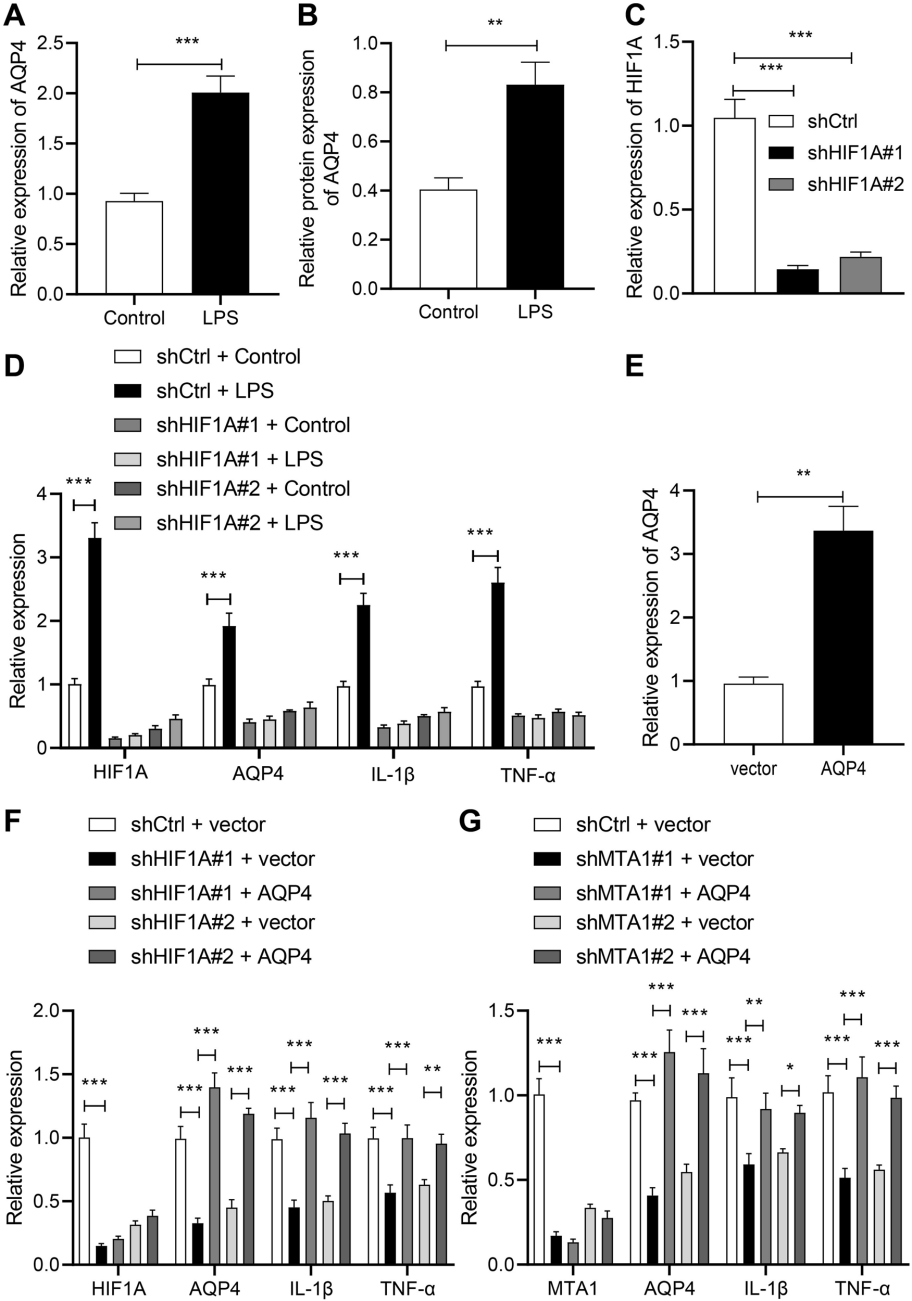
HIF1A promotes the occurrence of experimental colitis by increasing the expression pattern of AQP4. **A** The expression level of AQP4 in FHC cells treated with LPS determined with RT-qPCR. **B** Western blot analysis of the protein level of AQP4 in FHC cells treated with LPS. **C** RT-qPCR validation of the silencing efficiency of shHIF1A in FHC cells. **D** RT-qPCR measurement of the expression patterns of HIF1A, AQP4, IL-1β, and TNF-α in LPS-treated FHC cells with silencing of HIF1A. **E** RT-qPCR validation of the overexpression efficiency of AQP4 overexpression plasmid in FHC cells. **F** The expression patterns of HIF1A, AQP4, IL-1β, and TNF-α in LPS-treated cells with silencing of HIF1A detected by RT-qPCR. **G** The expression patterns of HIF1A, AQP4, IL-1β, and TNF-α in LPS-treated cells after MTA1 silencing. *p < 0.05; **p < 0.01; ***p < 0.001. The measurement data were expressed as mean ± standard deviation. Data between two groups were compared by the unpaired student’s *t* test, and data among multiple groups were compared by one-way ANOVA, followed by Tukey’s post hoc tests. The experiment was conducted three times independently.

In addition, LPS treatment up-regulated AQP4 mRNA and protein levels (Fig. 4A, B; Supplementary Fig. 1G). Thereafter, we silenced HIF1A in FHC cells and observed that sh-HIF1A#1 exhibited superior silencing effect, and thus was chosen for subsequent experimentation (Fig. 4C). Moreover, reduced levels of AQP4, IL-1β, and TNF-α transcription levels in response to sh-HIF1A#1 and sh-HIF1A#2 were observed (Fig. 4D). AQP4 overexpression could annul the down-regulation of IL-1β and TNF-α levels induced by HIF1A silencing (sh-HIF1A#1 or sh-HIF1A#2) in LPS-induced FHC cells (Fig. 4E, F). Moreover, following LPS treatment, AQP4 overexpression could evert down-regulation of IL-1β and TNF-α levels caused by MTA1 silencing (sh-MTA1#1 or sh-MTA1#2) in FHC cells (Fig. 4G). Besides, we performed identical experiments on NCM460 cells, and documented similar results (Supplementary Fig. 4A-G).

### Activated MTA1/HIF1A/AQP4 axis promotes apoptosis of colon epithelial cells

**Fig. 5:**
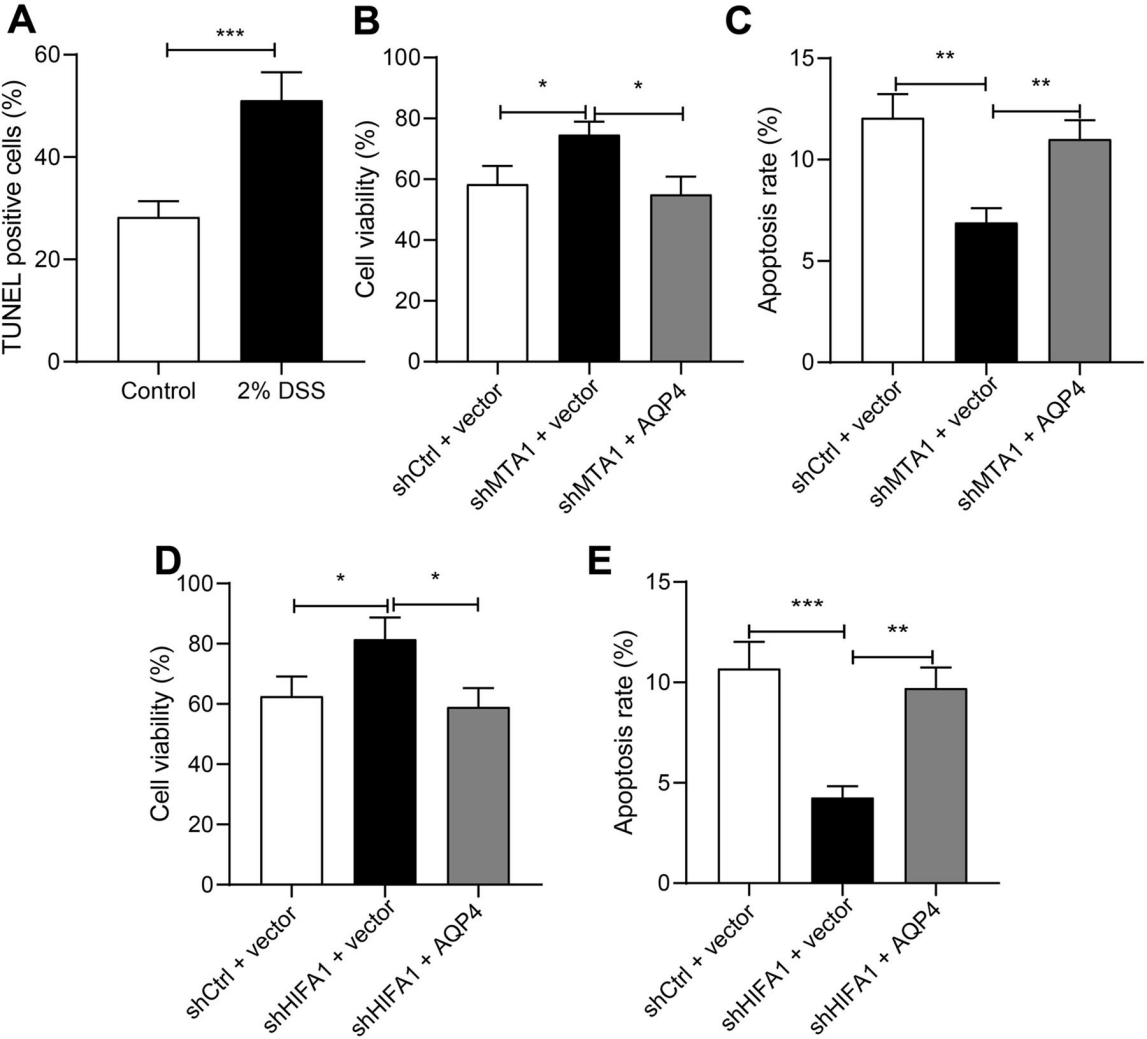
MTA1/HIF1A/AQP4 promotes the apoptosis of colon epithelial cells. **A** TUNEL staining of the cell apoptosis of colon tissue in DSS-induced experimental colitis. **B** The cell viability rate detected by CCK-8 assay in LPS-treated cells with MTA1 silencing. **C** The apoptotic rate detected by flow cytometry in LPS-treated cells with MTA1 silencing. **D** The cell viability rate detected by CCK-8 assay after AQP4 was overexpressed in LPS-treated cells with MTA1 silencing. **E** The apoptotic rate detected by flow cytometry after AQP4 was overexpressed in LPS-treated cells with MTA1 silencing. *p < 0.05; **p < 0.01; ***p < 0.001. The measurement data were expressed as mean ± standard deviation. Data between two groups were compared by the unpaired student’s *t* test, and data among multiple groups were compared by one-way ANOVA, followed by Tukey’s post hoc tests. The experiment was conducted three times independently.

Experimental mice presented with increased DSS-induced apoptosis (Fig. 5A). Subsequent results demonstrated that DSS-induced apoptotic rate of colon epithelial cells was reduced, and cell viability rate was augmented in mice by silencing HIF1A or MTA1, while being annulled by overexpression of AQP4 (Fig. 5B–E). Besides, we performed identical experiments on NCM460 cells, and documented similar results (Supplementary Fig. 5A–E).

### AQP4 inhibitor TGN-020 alleviates experimental colitis in mice

**Fig. 6:**
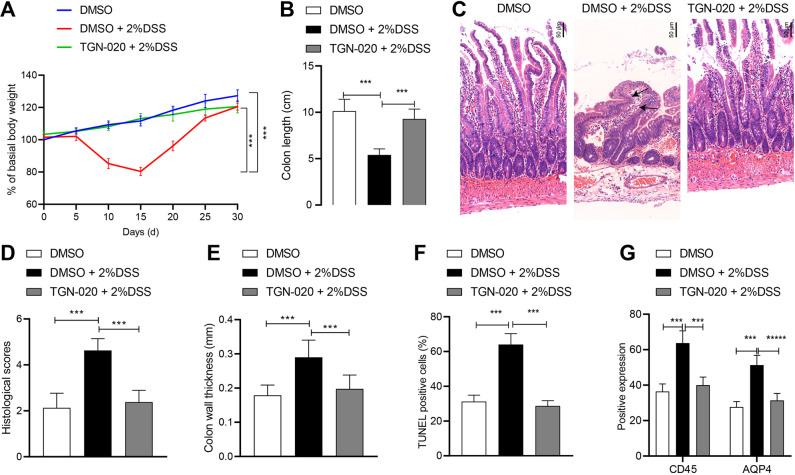
AQP4 inhibitor TGN-020 alleviates experimental colitis in mice. **A** The statistic analysis for the changes in mouse weight after treatment with TGN-020. **B** The colon length of the mice after treatment with TGN-020. **C** HE staining to observe the pathological changes of colon tissues in TGN-020 treated mice (200×). **D** Statistical scoring results. **E** Graphical quantification of the colon wall thickness. **F** TUNEL staining analysis the apoptosis of colon epithelial cells in TGN-020 treated mice. **G** Immunohistochemistry of the expression of CD45 and AQP4. *p < 0.05. The measurement data were expressed as mean ± standard deviation. Data between two groups were compared by the unpaired student’s *t* test, and data among multiple groups were compared by one-way ANOVA, followed by Tukey’s post hoc tests. The experiment was conducted three times independently. *n* = 8; ***p < 0.001.

Following DSS treatment on TGN-020-treated mice, TGN-020 alleviated weight loss and colon shortening of mice after DSS treatment (Fig. 6A, B). Meanwhile, compared to DMSO, treatment with DSS resulted in thickening of the colon wall, as evidenced by a large concentration of immune cell infiltration in mice; meanwhile, TGN-020 alleviated the aforementioned findings, with thinner colon wall and lower concentration of immune cell infiltration compared with DSS treatment (Fig. 6C–E). In addition, TGN-020 reduced degree of apoptosis of colon epithelial cells in DSS-induced mice (Fig. 6F). The positive rates of CD45 and AQP4 in the colon tissue of TGN-020-treated mice were decreased (Fig. 6G).

## Discussion

Initial experimentation illustrated that MTA1 was highly-expressed in DSS-induced experimental colitis mouse models. Despite the lack of research regarding the role of MTA1 in experimental colitis, a number of studies have documented the involvement of MTA1 in colorectal cancer and inflammatory responses. For instance, MTA1, as a direct target of miR-421, was previously illustrated to manipulate the cell progression of colorectal cancer [19]. Moreover, a prior study determined that activated MTA1 during inflammatory responses could mediate inflammation in co-ordination with transglutaminase 2, while MTA1 is also known to stimulate inflammation induced by monosodium urate crystal [20, 21]. Together, these findings shed a light on involvement of MTA1 in experimental colitis.

In addition, MTA1 facilitated experimental colitis *via* up-regulating HIF1A. Similarly, numerous studies have come across interaction between MTA1 and HIF1A under various conditions. One particular study identified that up-regulation of MTA1 elevated HIF1A in trophoblasts under hypoxic conditions, and could further serve as mediator of trophoblast function as well as differentiation in an early pregnancy [22]. Furthermore, MTA1 is known to improve stability of HIF1A protein by participation recruitment of histone deacetylase 1 [23]. What’s more, accumulating evidences have elicited the stimulative role of HIF1A in experimental colitis. For instance, stabilization of HIF1A by NIX, which was highly-expressed in the intestinal epithelium of ulcerative colitis patients, was previously uncovered to facilitate the aggravation of mitochondrial damage in intestinal inflammation [24]. Moreover, another study demonstrated that repression of the Ras-PI3K-Akt-HIF1A axis by treatment with the Huangqin Decoction could alleviate ulcerative colitis induced by DSS in mouse models, which is in accordance with our findings [25]. The Huangqin Decoction is primarily composed of baicalin, liquiritin, berberine, palmatine, and glycyrrhetinic acid, while as its chief component, baicalin possesses the ability to attenuate hypoxia-exposed H9c2 cell apoptosis by elevating HIF1α [26, 27]. Baicalin is further known to reduce the levels of AQP4, TNF-α, and IL-1β in rats with subarachnoid hemorrhage [28]. Strikingly, a prior study also documented that Berberine could suppress HIF1α and inhibits the radio-resistance of prostate cancer [29]. Meanwhile, increased expressions of HIF1A were previously identified in the colon of DSS-induced ulcerative colitis mice, which reiterates the significance of our findings [30].

Furthermore, another vital discovery was that HIF1A induced transcription of AQP4, which exacerbated the habitual occurrence of experimental colitis. Thanks to the hard-done work of our peers, the role of AQP4 in experimental colitis is well-established. For instance, AQP4 was previously identified as a significantly up-regulated gene in experimental colitis [31]. Moreover, deficiency in AQP4 has been established to result in the amelioration of experimental colitis in mouse models [13]. In addition, a plethora of studies have determined the relationship between HIF1A and AQP4. Expanding on the same, one particular study came across the ability of HIF1A to regulate the metabolism of its downstream marker AQP4 in edema formation and bioenergetics [32]. Further in line with our findings, pharmacological suppression of HIF1A due to 2-methoxyestradiol is known to induce a decline in the expression of AQP4 following traumatic brain injury [33].

**Fig. 7:**
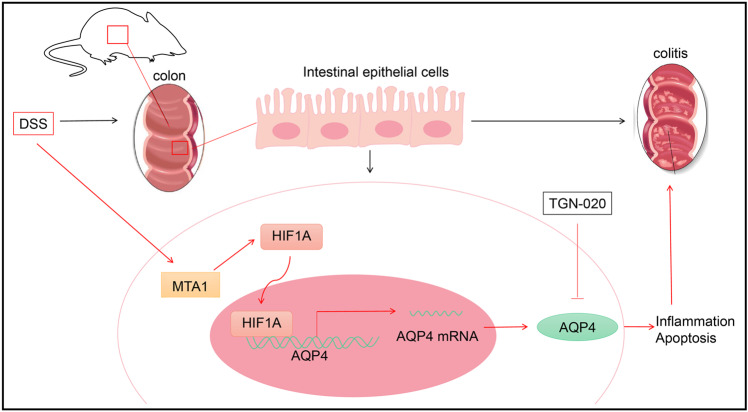
The molecular mechanism of the abnormal expression pattern of AQP4 in the development of experimental colitis in mice. MTA1 can activate HIF1A to induce the binding of HIF1A in the promoter region of AQP4, and thus promote the expression pattern of AQP4, thereby promoting the occurrence of inflammatory response and apoptosis of epithelial cells. Moreover, based on the molecular mechanism, TGN-020, the selective inhibitor of AQP4, can elicit an alleviatory role in experimental colitis in mice.

In light of the aforementioned findings, MTA1 can facilitate the binding of HIF1A in the promoter region of AQP4, which augments expression of AQP4, and thus promotes epithelial cell inflammation and apoptosis in experimental colitis (Fig. 7).

## Materials and methods

### Ethical approval

The current study was conducted with the approval of the Ethics committee of the Northern Jiangsu People’s Hospital. Animal experimentation protocols were in compliance with the Guidelines for the care and use of laboratory animals issued by the National Institutes of Health.

### Cell culture and transfection

Human embryonic kidney cells HEK293T and human normal colonic epithelial cells FHC cells were procured from the ATCC (HEK293T: ACS-4500, FHC: crl-1831, www.atcc.org). In addition, human colonic epithelial cell line NCM460 was obtained from CGMCC. The obtained HEK293T and NCM460 cells were then cultured in DMEM, and the FHC cells were cultured with RPMI-1640 (Gibco) at 37 °C with 5% CO_2_ in air.

The cells were infected with the adenovirus containing the short hairpin RNA control (shCtrl; control for shRNAs), shMTA1#1, shMTA1#2, shAQP4#1, shAQP4#2, shHIF1A#1, shHIF1A#2. The AQP4 overexpression plasmid was obtained from Addgene (Cambridge, MA).

Afterward, FHC and NCM460 cells were treated with 1 μg/mL LPS (Sigma-Aldrich Chemical Company, St Louis, MO) for 24 h to induce an in vitro inflammatory response [34].

### Adenovirus packaging and injection

Prior to transfection, the target plasmid was treated with Pac I (generally 6 μg for a 60-mm cell disk). Subsequently, plasmids treated with 6 μg Pac I were transfected into HEK293T cells with PEI or other transfection reagents. After 8 h, 4 mL DMEM (10% citrate-buffered saline, 1% Pen/Strep) was added. The cells were then scraped from the bottle and transferred into a 50-mL conical tube 7 to 10 days after transfection. Following centrifugation, the cells were resuspended with 2.0 mL PBS. Afterward, the cells were preserved in liquid nitrogen, dissolved in a 37 °C water bath, and shaken vigorously. The preceding steps were performed 4 times. Later, the HEK293T cells were placed in a 60-mm culture disk with 50–70% cell confluence, and the virus-contained supernatant was added at a volume ratio of 30–50%. Significant cell lysing or CPE was evident 2–3 days after infection. When 1/3rd to 1/2 of the cells were free and floating, the virus was isolated 3–5 days after infection. The presence of recombinant adenovirus was subsequently validated by western blot analysis or by the addition of 5 μL viral supernatant with 10 μL PCR-grade Protease K, digestion at 55 °C for 1 h, boiling for 5 min, centrifugation, and PCR (1–2 mL). Viral supernatant was collected with a minimum concentration of 1 × 10^7^ infectious particles/mL virus. For further amplification, the obtained virus supernatant was adopted to infect the cells in the 100-mm culture disk, and the virus was isolated for subsequent infection of HEK293T cells in a 150-mm culture disk to prepare an appropriate concentration of the virus. Next, 15% CsCl and 40% CsCl were added to a Beckman centrifuge tube to prepare the CsCl gradient solution. Afterward, the virus supernatant enriched with virus particles was added to the CsCl gradient solution. Ultracentrifugation was then performed at 30000 rpm at 4 °C for 16 h. Two bands were identified after centrifugation. The band presenting with the higher position and weaker color was regarded the primarily empty adenovirus shells, which did not have the ability to infect; the required band with lower position and brighter color containing live virus particles was isolated with a 16 needle. Dialysis was subsequently performed in tris-buffered saline (TBS) for 1 h, followed by two regimens of dialysis in TBS containing 10% glycerol, for 1 h each time. The purified adenovirus was aliquoted into Eppendorf tubes. In the Eppendorf biophotometer, 1 μg viral protein was found to be equivalent to 4 × 10^9^ virus particles. Finally, the collected samples were stored at −80 °C.

### DSS-induced experimental colitis models in mice

Forty female BALB/c mice (aged 5–6 weeks) were provided by Shanghai SLAC Laboratory Animal Co., Ltd. (Shanghai, China). After 5 days of acclimatization, the mice were fed with (1) normal water, (2) 2% DSS solution, (3) dimethyl sulfoxide (DMSO), (4) DMSO + 2% DSS (fed with DMSO containing 2% DSS), and (5) TGN-020 + 2% DSS (fed with 2% DSS and intraperitoneally injected with TGN-020 at 250 mg/kg) once daily for a period of 7 days, following which the alternations in the weight of mice were continuously observed and recorded. After 30 days, the mice were euthanized and the colon tissues were harvested for subsequent analyses. The investigator was blinded to the group allocation during the experiment and/or when assessing the outcomes.

### HE staining

First, 2 cm severe inflammation colon tissue samples were fixed with 10% formaldehyde solution for 24 h, paraffin-embedded and sectioned (5 μm). After dewaxing with xylene, the sections were dehydrated with gradient alcohol, and then immersed in hematoxylin solution for 5 min, in 1% hydrochloric acid alcohol for 30 s, and in 0.5% eosin solution for 5 min, and finally dehydrated with gradient alcohol. Afterward, the sections were cleared, sealed, and observed under a microscope (DM4B; Leica Biosystems, Shanghai, China).

### Immunohistochemistry

Two centimeters of severe inflammatory colon tissue samples were fixed in 10% formalin solution, paraffin-embedded, and sectioned (5 μm). Immunohistochemistry was performed as previously described [35]. After conventional steps, the slices were then subjected to overnight incubation at 4 °C with rabbit anti-human against AQP4 (1:1000, ab90088, Abcam, Cambridge, UK) and CD45 (1:200, #13917, Cell Signaling Technologies [CST], Beverly, MA). The following day, the slices were incubated with secondary goat anti-rabbit antibody against immunoglobulin G (IgG) (ab6785, 1:1000, Abcam) for 20 min at 37 °C. Streptomyces ovalbumin working solution labeled with horseradish peroxidase (HRP) (0343-10000U, Imunbio, Beijing, China) was then added to the slices, followed by reaction at 37 °C for 20 min. After diaminobenzidine (DAB) (ST033, Whiga, Guangzhou, China) color development, hematoxylin (PT001, Shanghai Boogu Biotechnology Co., Ltd., Shanghai, China) was employed to counteract the slices.

### RT-qPCR

Total RNA content was extracted with instructions of TRIzol (Invitrogen, Carlsbad, CA) kit, followed by determination of RNA concentration. Reverse transcription was performed using cDNA reverse transcription kits (K1622, Yaanda Biotechnology Co., Ltd., Beijing, China). Fluorescent quantitative PCR was carried out with a fluorescence quantitative PCR instrument (ViiA 7, Daangene, Guangzhou, China). The primers were synthesized by Sangon Biotechnology (Shanghai, China) (Supplementary Table 2). GAPDH was regarded as a normalizer, and relative transcription levels of target gene were calculated using 2^−△△Ct^ method.

### Western blot assay

Trypsin digestion was carried out to isolate the cultured cells or cut tissues, after which enhanced radioimmunoprecipitation assay lysis containing protease inhibitor (Boster, Wuhan, China) was added for ultrasonic lysing. Subsequently, bicinchoninic acid protein quantitative kits (Boster) were adopted to determine protein concentration. Next, the proteins were separated by 10% sodium dodecyl sulfate (SDS)-polyacrylamide gel electrophoresis and transferred onto a polyvinylidene fluoride membrane. After blocking, diluted primary antibodies against AQP4 (1:1000, #59678, CST), HIF1A (1:1000, #36169, CST), MTA1 (1:500, ab71153, Abcam), and GAPDH (1:1000, #5174, CST) were subsequently used to probe the membrane at 4 °C overnight. The following day, the membrane was re-probed with the HRP-labeled secondary antibody (goat anti-rabbit proteintech; SA00001-1; goat anti-mouse proteintech; SA00001-2) for 1 h. Enhanced chemiluminescence working solution (Millipore Corp., Billerica, MA) was added for culture for 1 min. Later, the membrane was placed in the Bio-rad automatic imager for exposure. Image Lab software was applied to quantify the gray levels.

### Dual-luciferase reporter gene assay

Following restriction endonuclease digestion, the AQP4 fragment containing HIF1A WT or MUT binding sites was inserted into the pGL3 vector reporter plasmid with the help of T4 DNA ligase to obtain pGL3-AQP4-WT or pGL3-AQP4-MUT. HIF1A overexpression vector or control vector was, respectively, co-transfected in cells for 36 h. Subsequently, the growth medium was completely aspirated, followed by the addition of 1 × passive lysis buffer for complete lysis. A dual-luciferase reporter system (E910, Promega Corporation, Madison, WI) and luciferase substrate luminescent detector were adopted for detection. All steps were carried out in strict accordance with the product manual and the recommended procedures in the operation manual.

### ChIP-qPCR

The cells were fixed with 1% formaldehyde at 37 °C for 10 min, and then rinsed twice with cold phosphate containing several protease inhibitors (1 mM phenylmethylsulfonyl fluoride, 1 μg/mL aprotinin, and 1 μg/mL pepsin A). Subsequently, the cells were incubated in pH 8.1 lysis buffer comprising of 1% SDS, 10 mM ethylene diamine tetraacetic acid (EDTA), and 50 mM Tris-HCl for 10 min, after which the genomic DNA was sliced using ultrasound. Following 10-min centrifugation of lysate at 13,000 r/min and 4 °C, the supernatant was diluted with a variety of reagents, including 0.01% SDS, 1% Triton X-100, 2 mM EDTA, 16.7 mM Tris-HCl, 167 mM NaCl (pH 8.1) and protease inhibitor. Anti-HIF1A (AF1935, R&D Systems, Minneapolis, MN) antibody was then supplemented to the lysate and cultured overnight at 4 °C. The following day, DNA fragments were recovered and PCR amplification was performed with the specific primers to detect the degree of enrichment of AQP4 promoter fragment binding to HIF1A.

### TUNEL staining

In strict accordance with the manufacturer’s instructions, the DeadEnd Colorimetric TUNEL System (Promega) was employed to evaluate the degree of apoptosis of colonic mucosal epithelial cells. Briefly, the DAB chromogenic solution was added to the samples for 3–6 min to facilitate color development. Following hematoxylin counterstaining, the cell apoptosis was observed under an optical microscope.

### Cell viability test

FHC cells were seeded in 96-well plates, at 1 × 10^3^ cells/well. In compliance with the provided instructions, the CCK-8 reagent (Dojindo Laboratories, Kumamoto, Japan) was adopted to measure the cell survival rate at 48 h. The optical density (OD) value was documented with an ELISA instrument (Bio-Rad Laboratories, Hercules, CA) to calculate the relative cell survival rate.

### Apoptosis detection

Briefly, 1 × 10^5^ treated FHC cells were attained. Apoptosis was subsequently detected using an Annexin V-fluorescein isothiocyanate/propidium iodide (PI) apoptosis detection kit (Beyotime, Shanghai, China), and the stained cells were identified with a flow cytometer. Annexin V-positive and PI-negative cells were regarded as apoptotic cells. The apoptosis rate was calculated using FlowJo software (Treestar, San Carlos, CA).

### Statistical analysis

Data analyses were performed using SPSS 21.0. Measurement data were summarized as mean ± standard deviation. Data between two groups were compared by unpaired *t* test, and those among multiple groups by one-way ANOVA or repeated measures of ANOVA, followed by Tukey’s post hoc tests. *p* < 0.05 was regarded as statistically significant.

## Supplementary information

Figrue S1

Figrue S2

Figrue S3

Figrue S4

Figrue S5

Supplementary Figure Legends

Supplementary Tables

author-contribution-form

## Data Availability

The datasets generated and/or analyzed during the current study are available from the corresponding author on reasonable request.
