# Supplementary figures and images for: MTA1 aggravates experimental colitis in mice by promoting transcription factor HIF1A and up-regulating AQP4 expression
Source: Cell Death Discov. 2022 Jun 28;8:298. doi: 10.1038/s41420-022-01052-y (PMC9240051; doi:10.1038/s41420-022-01052-y)

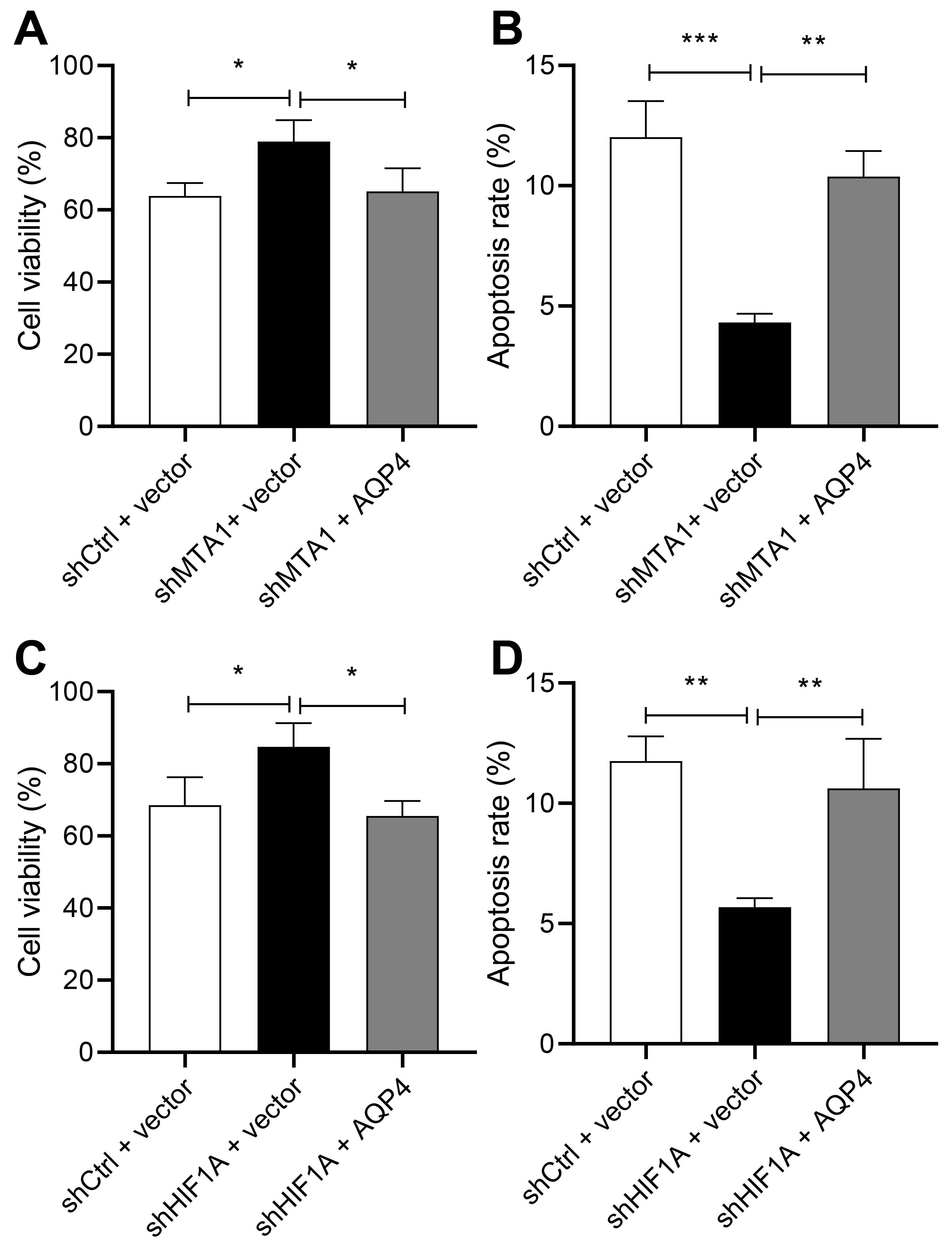

Supplement: Supplementary file 5 — Figrue S5 [file 41420_2022_1052_MOESM5_ESM.tif]
