# Supplementary material for: MTA1 aggravates experimental colitis in mice by promoting transcription factor HIF1A and up-regulating AQP4 expression
Source: Cell Death Discov. 2022 Jun 28;8:298. doi: 10.1038/s41420-022-01052-y (PMC9240051; doi:10.1038/s41420-022-01052-y)
Supplement: Supplementary file 6 — Supplementary Figure Legends [file 41420_2022_1052_MOESM6_ESM.docx]

**Supplementary Figure 1** Western blots. A, The protein expression pattern of MTA1 in tissues from control mice and 2% DSS-treated mice detected by Western blot, n = 8. B, The protein expression pattern of MTA1 detected by Western blot. C, Western blot to detect the protein expression of HIF1A in colon tissues after DSS-induced experimental colitis in mice, n = 8. D, The protein expression pattern of HIF1A in FHC cells induced by LPS detected by Western blot. E, The protein expression pattern of HIF1A in MTA1 silenced FHC cells treated with LPS detected by Western blot. F, The expression pattern of AQP4 was detected by Western blot after MTA1 silenced FHC cells were treated with LPS. G, Western blot analysis of the protein level of AQP4 in FHC cells treated with LPS.

**Supplementary Figure 2** MTA1 promotes the inflammation in NCM460 cells. A, RT-qPCR to detect the mRNA expression patterns of IL-1β and TNF-α in LPS-treated NCM460 cells. B, The protein expression pattern of MTA1 in LPS-treated NCM460 cells detected by Western blot. C, The expression pattern of MTA1 mRNA in LPS-treated NCM460 cells detected by RT-qPCR. D, RT-qPCR to detect sh-MTA1 silencing efficiency in NCM460 cells. E, The expression patterns of MTA1, IL-1β and TNF-α in NCM460 cells treated with LPS determined with RT-qPCR. * *p* < 0.05; ***p* < 0.01; ****p* < 0.001. The measurement data were expressed as mean ± standard deviation. Data between two groups were compared by the unpaired student’s *t* test, and data among multiple groups were compared by one-way ANOVA, followed by Tukey’s post hoc tests. The experiment was conducted three times independently.

**Supplementary Figure 3** MTA1 upregulates the expression pattern of HIF1A to promote its transcriptional regulation of AQP4 in NCM460 cells. A, The protein expression pattern of AQP4 was detected by Western blot after MTA1 silenced NCM460 cells were treated with LPS. B, RT-qPCR validation of overexpression efficiency of MTA1 overexpression plasmid in NCM460 cells. C, The effect of MTA1 overexpression in the enrichment of HIF1A in the promoter region of AQP4 IN NCM460 CELLS determined with ChIP assay. ***p* < 0.01; ****p* < 0.001. The measurement data were expressed as mean ± standard deviation. Data between two groups were compared by the unpaired student’s *t* test, and data among multiple groups were compared by one-way ANOVA, followed by Tukey’s post hoc tests. The experiment was conducted three times independently.

**Supplementary Figure 4** HIF1A promotes the occurrence of experimental colitis by increasing the expression pattern of AQP4 in the NCM460 cells. A, The expression level of AQP4 in NCM460 cells treated with LPS determined with RT-qPCR. B, Western blot analysis of the protein level of AQP4 in NCM460 cells treated with LPS. C, RT-qPCR validation of the silencing efficiency of shHIF1A in NCM460 cells. D, RT-qPCR measurement of the expression patterns of HIF1A, AQP4, IL-1β and TNF-α in LPS-treated NCM460 cells with silencing of HIF1A. E. RT-qPCR validation the overexpression efficiency of AQP4 overexpression plasmid in NCM460 cells. F, The expression patterns of HIF1A, AQP4, IL-1β and TNF-α in LPS-treated NCM460 cells with silencing of HIF1A detected by RT-qPCR. G, The expression patterns of HIF1A, AQP4, IL-1β and TNF-α in LPS-treated NCM460 cells after MTA1 silencing. * *p* < 0.05; ***p* < 0.01; ****p* < 0.001. The measurement data were expressed as mean ± standard deviation. Data between two groups were compared by the unpaired student’s *t* test, and data among multiple groups were compared by one-way ANOVA, followed by Tukey’s post hoc tests. The experiment was conducted three times independently.

**Supplementary Figure 5** MTA1/HIF1A/AQP4 promotes the apoptosis of colon epithelial cells. A, The cell viability rate detected by CCK-8 assay in LPS-treated NCM460 cells with MTA1 silencing. B, The apoptotic rate detected by flow cytometry in LPS-treated NCM460 cells with MTA1 silencing. C, The cell viability rate detected by CCK-8 assay after AQP4 was overexpressed in LPS-treated NCM460 cells with MTA1 silencing. D, The apoptotic detected by flow cytometry after AQP4 was overexpressed in LPS-treated NCM460 cells with MTA1 silencing. * *p* < 0.05; ***p* < 0.01; ****p* < 0.001. The measurement data were expressed as mean ± standard deviation. Data among multiple groups were compared by one-way ANOVA, followed by Tukey’s post hoc tests. The experiment was conducted three times independently.
