# Supplementary material for: MTA1 aggravates experimental colitis in mice by promoting transcription factor HIF1A and up-regulating AQP4 expression
Source: Cell Death Discov. 2022 Jun 28;8:298. doi: 10.1038/s41420-022-01052-y (PMC9240051; doi:10.1038/s41420-022-01052-y)
Supplement: Supplementary file 7 — Supplementary Tables [file 41420_2022_1052_MOESM7_ESM.docx]

**Supplementary Table 1** AQP4 and its interaction gene enrichment pathways

| Term | ID | Input_number | Background_number | P_Value | Corrected P-Value | Input | Hyperlink |
| --- | --- | --- | --- | --- | --- | --- | --- |
| Mitophagy - animal | hsa04137 | 2 | 65 | 0.000772546 | 0.010181057 | CSNK2A2\|CSNK2A1 | http://www.genome.jp/kegg-bin/show_pathway?hsa04137/hsa:1459%09red |
| Adherens junction | hsa04520 | 2 | 72 | 0.000941297 | 0.011577956 | CSNK2A2\|CSNK2A1 | http://www.genome.jp/kegg-bin/show_pathway?hsa04520/hsa:1459%09red |
| PD-L1 expression and PD-1 checkpoint pathway in cancer | hsa05235 | 2 | 89 | 0.001418091 | 0.014535431 | CSNK2A2\|CSNK2A1 | http://www.genome.jp/kegg-bin/show_pathway?hsa05235/hsa:1459%09red |
| Inflammatory mediator regulation of TRP channels | hsa04750 | 2 | 100 | 0.001776485 | 0.016388078 | PLA2G6\|TRPV4 | http://www.genome.jp/kegg-bin/show_pathway?hsa04750/hsa:8398%09red/hsa:59341%09red |
| NF-kappa B signaling pathway | hsa04064 | 2 | 100 | 0.001776485 | 0.016388078 | CSNK2A2\|CSNK2A1 | http://www.genome.jp/kegg-bin/show_pathway?hsa04064/hsa:1459%09red |
| Ribosome biogenesis in eukaryotes | hsa03008 | 2 | 105 | 0.001952184 | 0.017569659 | CSNK2A2\|CSNK2A1 | http://www.genome.jp/kegg-bin/show_pathway?hsa03008/hsa:1459%09red |
| Measles | hsa05162 | 2 | 138 | 0.003308552 | 0.022901405 | CSNK2A2\|CSNK2A1 | http://www.genome.jp/kegg-bin/show_pathway?hsa05162/hsa:1459%09red |
| Wnt signaling pathway | hsa04310 | 2 | 160 | 0.004398266 | 0.023691086 | CSNK2A2\|CSNK2A1 | http://www.genome.jp/kegg-bin/show_pathway?hsa04310/hsa:1459%09red |
| alpha-Linolenic acid metabolism | hsa00592 | 1 | 25 | 0.015774974 | 0.036841554 | PLA2G6 | http://www.genome.jp/kegg-bin/show_pathway?hsa00592/hsa:8398%09red |
| Linoleic acid metabolism | hsa00591 | 1 | 29 | 0.018180626 | 0.039462652 | PLA2G6 | http://www.genome.jp/kegg-bin/show_pathway?hsa00591/hsa:8398%09red |
| SNARE interactions in vesicular transport | hsa04130 | 1 | 34 | 0.021179766 | 0.043661082 | STX5 | http://www.genome.jp/kegg-bin/show_pathway?hsa04130/hsa:6811%09red |
| Vasopressin-regulated water reabsorption | hsa04962 | 1 | 44 | 0.027151729 | 0.05060095 | AQP4 | http://www.genome.jp/kegg-bin/show_pathway?hsa04962/hsa:361%09red |
| Proteasome | hsa03050 | 1 | 45 | 0.027747001 | 0.051193218 | PSMD4 | http://www.genome.jp/kegg-bin/show_pathway?hsa03050/hsa:5710%09red |
| Ether lipid metabolism | hsa00565 | 1 | 47 | 0.028936499 | 0.052859248 | PLA2G6 | http://www.genome.jp/kegg-bin/show_pathway?hsa00565/hsa:8398%09red |
| Endocrine and other factor-regulated calcium reabsorption | hsa04961 | 1 | 50 | 0.03071813 | 0.054758406 | AP2M1 | http://www.genome.jp/kegg-bin/show_pathway?hsa04961/hsa:1173%09red |
| N-Glycan biosynthesis | hsa00510 | 1 | 50 | 0.03071813 | 0.054758406 | DOLPP1 | http://www.genome.jp/kegg-bin/show_pathway?hsa00510/hsa:57171%09red |
| Arachidonic acid metabolism | hsa00590 | 1 | 63 | 0.038402398 | 0.061610804 | PLA2G6 | http://www.genome.jp/kegg-bin/show_pathway?hsa00590/hsa:8398%09red |
| Bile secretion | hsa04976 | 1 | 72 | 0.043688039 | 0.066341097 | AQP4 | http://www.genome.jp/kegg-bin/show_pathway?hsa04976/hsa:361%09red |
| Synaptic vesicle cycle | hsa04721 | 1 | 78 | 0.04719632 | 0.069108897 | AP2M1 | http://www.genome.jp/kegg-bin/show_pathway?hsa04721/hsa:1173%09red |
| Fc gamma R-mediated phagocytosis | hsa04666 | 1 | 94 | 0.056491537 | 0.076637417 | PLA2G6 | http://www.genome.jp/kegg-bin/show_pathway?hsa04666/hsa:8398%09red |
| Glycerophospholipid metabolism | hsa00564 | 1 | 97 | 0.058224689 | 0.076942686 | PLA2G6 | http://www.genome.jp/kegg-bin/show_pathway?hsa00564/hsa:8398%09red |
| Lysosome | hsa04142 | 1 | 123 | 0.073118067 | 0.090843659 | AP3M1 | http://www.genome.jp/kegg-bin/show_pathway?hsa04142/hsa:26985%09red |
| Autophagy - animal | hsa04140 | 1 | 128 | 0.075956183 | 0.093426105 | VMP1 | http://www.genome.jp/kegg-bin/show_pathway?hsa04140/hsa:81671%09red |
| Vascular smooth muscle contraction | hsa04270 | 1 | 132 | 0.078220677 | 0.095891793 | PLA2G6 | http://www.genome.jp/kegg-bin/show_pathway?hsa04270/hsa:8398%09red |
| Ubiquitin mediated proteolysis | hsa04120 | 1 | 137 | 0.081043817 | 0.098372264 | STUB1 | http://www.genome.jp/kegg-bin/show_pathway?hsa04120/hsa:10273%09red |
| Fluid shear stress and atherosclerosis | hsa05418 | 1 | 139 | 0.082170751 | 0.099088258 | TRPV4 | http://www.genome.jp/kegg-bin/show_pathway?hsa05418/hsa:59341%09red |
| Cellular senescence | hsa04218 | 1 | 160 | 0.093923879 | 0.110728151 | TRPV4 | http://www.genome.jp/kegg-bin/show_pathway?hsa04218/hsa:59341%09red |
| Protein processing in endoplasmic reticulum | hsa04141 | 1 | 165 | 0.096700909 | 0.113638966 | STUB1 | http://www.genome.jp/kegg-bin/show_pathway?hsa04141/hsa:10273%09red |
| Huntington disease | hsa05016 | 1 | 193 | 0.11210217 | 0.127279079 | AP2M1 | http://www.genome.jp/kegg-bin/show_pathway?hsa05016/hsa:1173%09red |
| Epstein-Barr virus infection | hsa05169 | 1 | 201 | 0.116456102 | 0.131816876 | PSMD4 | http://www.genome.jp/kegg-bin/show_pathway?hsa05169/hsa:5710%09red |
| Ras signaling pathway | hsa04014 | 1 | 232 | 0.133135126 | 0.147086412 | PLA2G6 | http://www.genome.jp/kegg-bin/show_pathway?hsa04014/hsa:8398%09red |
| Endocytosis | hsa04144 | 1 | 244 | 0.139510162 | 0.152757417 | AP2M1 | http://www.genome.jp/kegg-bin/show_pathway?hsa04144/hsa:1173%09red |
| Metabolic pathways | hsa01100 | 1 | 1433 | 0.590618975 | 0.593837607 | PLA2G6 | http://www.genome.jp/kegg-bin/show_pathway?hsa01100/hsa:8398%09red |

**Supplementary Table 2** Primer sequences for RT-qPCR

| Gene | Sequence |
| --- | --- |
| m. AQP4 | F: 5’-ATCAGCATCGCTAAGTCCGTC-3’ |
|  | R: 5’-GAGGTGTGACCAGGTAGAGGA-3’ |
| m. HIF1A | F: 5’-TCTCGGCGAAGCAAAGAGTC-3’ |
|  | R: 5’-AGCCATCTAGGGCTTTCAGATAA-3’ |
| m. MTA1 | F: 5’-CAACCCGTACCTGATCCGC-3’ |
|  | R: 5’-GCCTCCACATTCCCGTTGG-3’ |
| h. AQP4 | F: 5’-AGCAGTCACAGCGGAATTTCT-3’ |
|  | R: 5’-TCTGTTCCACCCCAGTTGATG-3’ |
| h. HIF1A | F: 5’-GAACGTCGAAAAGAAAAGTCTCG-3’ |
|  | R: 5’-CCTTATCAAGATGCGAACTCACA-3’ |
| h. MTA1 | F: 5’-ACGCAACCCTGTCAGTCTG-3’ |
|  | R: 5’-GGGCAGGTCCACCATTTCC-3’ |
| h. IL-1β | F: 5’-CCACAGACCTTCCAGGAGAATG-3’ |
|  | R: 5’-GTGCAGTTCAGTGATCGTACAGG-3’ |
| h. TNF-α | F: 5’-CTCTTCTGCCTGCTGCACTTTG-3’ |
|  | R: 5’-ATGGGCTACAGGCTTGTCACTC-3’ |
| m. Actin | F: 5’-GTGACGTTGACATCCGTAAAGA-3’ |
|  | R: 5’-GCCGGACTCATCGTACTCC-3’ |
| h. Actin | F: 5’-CATGTACGTTGCTATCCAGGC-3’ |
|  | R: 5’-CTCCTTAATGTCACGCACGAT-3’ |

Note: AQP4, aquaporin 4; HIF1A, hypoxia-inducible factor 1α; MTA1, metastasis associated protein 1; IL-1β, interleukin-1β; TNF-α, tumor necrosis factor-α; RT-qPCR, reverse transcription-quantitative polymerase chain reaction; m, mouse; h, human.
