# Supplementary material for: MTA1 aggravates experimental colitis in mice by promoting transcription factor HIF1A and up-regulating AQP4 expression
Source: Cell Death Discov. 2022 Jun 28;8:298. doi: 10.1038/s41420-022-01052-y (PMC9240051; doi:10.1038/s41420-022-01052-y)
Supplement: Supplementary file 8 — author-contribution-form [file 41420_2022_1052_MOESM8_ESM.pdf]

DECLARATION OF CONTRIBUTIONS TO ARTICLE

ADMC

Manuscript Number: CDDISCOVERY-21-1682

Journal Name: *Cell Death Discovery* (the 'Journal')

Proposed Title of the Contribution:

MTA1 aggravates experimental colitis in mice by promoting transcription factor HIF1A to upregulate AQP4 expression (the 'Contribution)

Author(s):

Ping Li, Dong-Ping Shi, Tao Jin, Dong Tang, Wei Wang and Liu-Hua Wang (the 'Authors')

For all *CDDis*covery articles, each person named as an author in the published version must be able to show he or she has contributed substantially to the article.

Authorship credit should be based on 1) substantial contributions to conception and design, acquisition of data, or analysis and interpretation of data; 2) drafting the article or revising it critically for important intellectual content; and 3) final approval of the version to be published. Authors should meet conditions 1, 2 and 3.

Any person who cannot be shown to have made a substantial contribution to the article cannot be listed as an author in the final version. The name of any person who is deemed to have made a minor contribution can, however, appear in the Acknowledgments section of the article.

Please complete the table below to indicate the contributions of all named authors to the manuscript.

Author Full Name:	Specification of Contribution to the Manuscript:
Ping Li	Conceptualization; Methodology; Writing—original draft preparation; Writing—review and editing
Dong-Ping Shi	Conceptualization; Methodology; Writing—original draft preparation; Writing—review and editing
Tao Jin	Methodology; Data analysis; Writing—review and editing; Supervision
Dong Tang	Software; Validation; Data analysis; Writing—review and editing
Wei Wang	Validation; Writing—review and editing; Supervision
Liu-Hua Wang	Validation; Writing—review and editing; Project administration

Please complete the table below to indicate the contributions of all named authors to the figures.

Figure 1:

Tao Jin, Dong Tang, Wei Wang, Liu-Hua Wang

Figure 2:

Tao Jin, Dong Tang, Wei Wang, Liu-Hua Wang

Figure 3:

Tao Jin, Dong Tang, Wei Wang, Liu-Hua Wang

Figure 4:

Tao Jin, Dong Tang, Wei Wang, Liu-Hua Wang

Figure 5:

Tao Jin, Dong Tang, Wei Wang, Liu-Hua Wang

Figure 6:

Tao Jin, Dong Tang, Wei Wang, Liu-Hua Wang

Signed for and on behalf of the Author(s):	Print Name:	Date:
Liu-Hua Wang	Liu-Hua Wang	2021/7/23
